# Association of Genetically Predicted BCAA Levels with Muscle Fiber Size in Athletes Consuming Protein

**DOI:** 10.3390/genes13030397

**Published:** 2022-02-23

**Authors:** Elliott C. R. Hall, Ekaterina A. Semenova, Elvira A. Bondareva, Liliya B. Andryushchenko, Andrey K. Larin, Pawel Cięszczyk, Edward V. Generozov, Ildus I. Ahmetov

**Affiliations:** 1 Research Institute for Sport and Exercise Sciences, Liverpool John Moores University, Liverpool L3 5AF, UK; elliotthall@live.co.uk; 2 Department of Molecular Biology and Genetics, Federal Research and Clinical Center of Physical-Chemical Medicine of Federal Medical Biological Agency, 119435 Moscow, Russia; alecsekaterina@gmail.com (E.A.S.); bondareva.e@gmail.com (E.A.B.); zelaz@yandex.ru (A.K.L.); generozov@gmail.com (E.V.G.); 3 Research Institute of Physical Culture and Sport, Volga Region State University of Physical Culture, Sport and Tourism, 420010 Kazan, Russia; 4 Department of Physical Education, Plekhanov Russian University of Economics, 115093 Moscow, Russia; andryushenko-lil@mail.ru; 5 Faculty of Physical Education, Gdańsk University of Physical Education and Sport, 80-854 Gdańsk, Poland; cieszczyk@poczta.onet.pl; 6 Laboratory of Molecular Genetics, Kazan State Medical University, 420012 Kazan, Russia

**Keywords:** genetics, DNA, polymorphism, genotype, muscle protein synthesis, anabolism, hypertrophy, endurance, athletes

## Abstract

Branched-chain amino acid (BCAA) levels are associated with skeletal muscle cross-sectional area (CSA). Serum BCAA levels are enhanced by whey protein supplementation (WPS), and evidence in clinical populations suggests an association of single nucleotide polymorphisms (SNPs) with BCAA metabolite levels. It is not known whether the same SNPs are associated with the ability to catabolise BCAAs from exogenous sources, such as WPS. The present study investigated whether possessing a higher number of alleles associated with increased BCAA metabolites correlates with muscle fiber CSA of m. vastus lateralis in physically active participants, and whether any relationship is enhanced by WPS. Endurance-trained participants (*n* = 75) were grouped by self-reported habitual WPS consumption and genotyped for five SNPs (*PPM1K* rs1440580, *APOA5* rs2072560, *CBLN1* rs1420601, *DDX19B* rs12325419, and *TRMT61A* rs58101275). Body mass, BMI, and fat percentage were significantly lower and muscle mass higher in the WPS group compared to Non-WPS. The number of BCAA-increasing alleles was correlated with fiber CSA in the WPS group (*r* = 0.75, *p* < 0.0001) and was stronger for fast-twitch fibers (*p* = 0.001) than slow-twitch fibers (*p* = 0.048). Similar results remained when corrected for multiple covariates (age, physical activity, and meat and dairy intake). No correlation was found in the Non-WPS group. This study presents novel evidence of a positive relationship between BCAA-increasing alleles and muscle fiber CSA in athletes habitually consuming WPS. We suggest that a high number of BCAA-increasing alleles improves the efficiency of WPS by stimulation of muscle protein synthesis, and contributes to greater fiber CSA.

## 1. Introduction

Essential amino acids (EAAs) stimulate skeletal muscle protein synthesis (MPS) [1], which is crucial for growth and repair [2]. Importantly, EAAs cannot be attained through de novo synthesis and must be acquired through nutritional sources, making dietary EAA provision critical for enhancing post-exercise MPS [3]. Of the EAAs, the branched-chain amino acids (BCAAs; leucine, isoleucine, and valine) initiate MPS by stimulating mRNA translation [1] and prevent muscle protein degradation (MPD) via inhibitory mechanisms relating to the mammalian target of rapamycin (mTOR) pathway [4]. Coupled with the ability of skeletal muscle to oxidise BCAAs for energy provision, many athletes view BCAA ingestion as an important strategy to augment post-exercise recovery [5] and delay perceived fatigue onset [6]. However, a direct impact on performance remains unclear [7,8].

Whey protein supplementation (WPS) is a popular nutritional strategy, principally due to a similar amino acid profile to human skeletal muscle [9], containing ~25% BCAAs [10]. Ingesting WPS increases serum BCAA levels, which are positively correlated with skeletal muscle index (skeletal muscle mass/height^2^), muscle cross-sectional area (CSA) [11,12], and fat-free mass (UK Biobank). Physically active individuals demonstrate greater capacity to utilise BCAAs than sedentary populations [13], which is purportedly linked to higher mRNA expression of BCAA catabolism pathways [14]. In sedentary populations, high serum BCAA concentrations are associated with metabolic risk factors including low physical activity, adiposity, and insulin resistance [15,16], suggesting exercise is important for BCAA catabolism. It appears that higher BCAA concentrations might be beneficial for active individuals, possibly due to the role of exercise in augmenting BCAA catabolism and, subsequently, stimulating MPS.

Genomic and metabolomic investigations exploring the mechanistic links between impaired BCAA catabolism and both type 2 diabetes (T2D) and metabolic syndrome [17] report single nucleotide polymorphisms (SNPs) associated with circulating levels of BCAA metabolites [18]. Specifically, genome-wide analysis of 16,596 individuals revealed that the alleles of several SNPs that were associated with increased T2D risk were also associated with greater metabolite concentrations of one or more BCAAs, suggesting those variants might impair BCAA catabolism [18]. However, BCAA-increasing alleles have also been associated with increased values of fat-free mass and levels of insulin-like growth factor 1 (IGF1) [19], supporting the association of BCAA levels with skeletal muscle index and muscle CSA [12] and suggesting that these alleles might also affect skeletal muscle phenotypes. To our knowledge, no study has investigated whether BCAA-increasing alleles are associated with skeletal muscle phenotypes in active populations, where the effect of exercise stimuli on BCAA catabolism pathways [14] may have different outcomes compared to sedentary populations.

Greater fat-free mass and IGF1 levels in carriers of BCAA-increasing alleles (UK Biobank) suggest some individuals have an increased capacity to utilise BCAAs for muscle repair and growth. It follows that carrying multiple BCAA-increasing alleles may augment the ability to catabolise and utilise the additional serum BCAAs provided by WPS for MPS enhancement. However, the relationship between WPS efficiency and BCAA-increasing alleles is unclear. Therefore, the aim of the present study was to investigate whether possessing a higher number of alleles previously associated with increased BCAA metabolites is related to muscle fiber CSA in endurance-trained participants, and whether any relationship is enhanced by WPS. We hypothesised that carrying a higher number of alleles associated with increased BCAA levels (*PPM1K* rs1440580 A, *APOA5* rs2072560 C, *CBLN1* rs1420601 C, *DDX19B* rs12325419 G, and *TRMT61A* rs58101275 G alleles, Table 1) would be positively related to muscle fiber CSA in endurance-trained participants, particularly those consuming WPS.

## 2. Materials and Methods

### 2.1. Ethical Approval

The study was approved by the Ethics Committee of the Federal Research and Clinical Center of Physical-chemical Medicine of the Federal Medical and Biological Agency of Russia (protocol #2017/04). Written informed consent was obtained from each participant. The study complied with the Declaration of Helsinki and ethical standards for sport and exercise science research.

### 2.2. Study Participants

A total of 75 endurance-trained males participated in this study. The types of endurance training included running, swimming, skating, rowing, cycling, and skiing. All participants were healthy (no history of chronic diseases; did not take any of medications). All participants were Caucasians of Eastern European ethnicity. Participants were divided into two groups depending on their self-reported habitual WPS consumption. Specifically, the WPS group (*n* = 22) consumed 20–25 g of whey protein per day for at least one year. The Non-WPS group (*n* = 53) had no history of protein supplementation. Participant characteristics are described in Table 2.

### 2.3. Anthropometry and Body Composition

Anthropometric measurements were taken according to standard techniques [20] using the GPM anthropometric measurement kit (DKSH, Switzerland). Subjects were measured barefoot, wearing only underwear. Body mass was measured using a battery-operated digital scale (100 g precision). Height was measured using an anthropometer (1 mm precision). Body composition analysis was performed in a sub-sample of participants (*n* = 39, Table 2). The whole-body impedance (body composition) was measured in a supine position on the right side of the body according to a conventional tetrapolar measurement scheme by the bioimpedance analyzer ABC-02 ‘Medas’ (SRC Medas, Moscow, Russia) at a frequency of 50 kHz using disposable Ag/AgCl FIAB (Italy) bioadhesive electrodes. Body composition measurements included fat mass (kg), fat percentage (%), and muscle mass (kg).

### 2.4. Physical Activity and Training Parameters

Participants were classified according to their training frequency as mildly active (2 training sessions per week), moderately active (3–4 training sessions per week), highly active (5–7 training sessions per week), or extremely active (two training sessions per day).

### 2.5. Assessment of Dietary Protein Intake

Dietary protein intake was assessed using a dietary questionnaire administered to all participants. Responses to the questions on meat and dairy were assigned values for frequency per week (never  =  0, once per week  =  1, 2–3 times per week  =  3, 4–5 times per week  =  5, and once or more a day  =  7).

### 2.6. Evaluation of Muscle Fiber Composition by Immunohistochemistry

Vastus lateralis samples were obtained from the left leg using the modified Bergström needle procedure [21] with aspiration under local anaesthesia using 2% lidocaine solution. Prior to analysis, samples were frozen in liquid nitrogen and stored at −80 °C. Serial cross-sections (7 μm) were obtained from frozen samples using an ultratom (Leica Microsystems, Wetzlar, Germany). Sections were thaw-mounted on Polysine glass slides, maintained at room temperature (RT) for 15 min and incubated in PBS (3 × 5 min). The sections were then incubated at RT in primary antibodies against slow or fast isoforms of the myosin heavy chains (M8421, 1:5000; M4276; 1:600, respectively; Sigma-Aldrich, Burlington, MA, USA) for 1 h and incubated in PBS (3 × 5 min). Afterwards, the sections were incubated at RT in secondary antibodies conjugated with FITC (F0257; 1:100; Sigma-Aldrich) for 1 h. The antibodies were removed and the sections washed in PBS (3 × 5 min), placed in mounting media and covered with a cover slip. Images were captured by fluorescent microscope (Eclipse Ti-U, Nikon, Tokyo, Japan). All analysed images contained 330 ± 11 fibers. The ratio of the number of stained fibers to the total fiber number was calculated. Fibers stained in serial sections with antibodies against slow and fast isoforms were considered hybrid fibers.

### 2.7. Genotyping

Molecular genetic analysis was performed with DNA samples obtained from leukocytes (venous blood). Four ml of venous blood were collected in tubes containing EDTA (Vacuette EDTA tubes, Greiner Bio-One, Kremsmünster, Austria). Blood samples were transported to the laboratory at 4 °C and DNA was extracted on the same day. DNA extraction and purification were performed using a commercial kit according to the manufacturer’s instructions (Technoclon, Moscow, Russia) and included chemical lysis, selective DNA binding on silica spin columns and ethanol washing. Extracted DNA quality was assessed by agarose gel electrophoresis at this step. HumanOmniExpress BeadChips (Illumina Inc, San Diego, CA, USA) were used for genotyping of >900,000 SNPs, including *PPM1K* rss1440580, *APOA5* rs2072560, *CBLN1* rs1420601, *DDX19B* rs12325419, and *TRMT61A* rs58101275. The assay required 200 ng of DNA sample as input with a concentration of at least 50 ng/µL. Exact concentrations of DNA in each sample were measured using a Qubit Fluorometer (Invitrogen, Waltham, MA, USA). All further procedures were performed according to the instructions of Infinium HD Assay.

### 2.8. Polygenic Analysis

A polygenic approach (i.e., the combined association of all 5 SNPs) was used to investigate the relationship between the five SNPs previously associated with BCAA levels and muscle fiber CSA. Based on existing evidence, participants were categorised according to the number of BCAA-increasing alleles they possessed. For example, carriers of *PPM1K* rs1440580 TT, *APOA5* rs2072560 TT, *CBLN1* rs1420601 TT, *DDX19B* rs12325419 AA, and *TRMT61A* rs58101275 AA genotypes had zero BCAA-increasing alleles, whereas participants with *PPM1K* rs1440580 AA, *APOA5* rs2072560 CC, *CBLN1* rs1420601 CC, *DDX19B* rs12325419 GG, and *TRMT61A* rs58101275 GG genotypes had 10 BCAA-increasing alleles. Heterozygous genotypes were computed with intermediate scores. 

### 2.9. Statistical Analyses

Statistical analyses were conducted using GraphPad InStat (GraphPad Software, Inc., San Diego, CA, USA) software. Differences in characteristics between two groups (i.e., non-WPS vs. WPS) were analysed using unpaired *t* tests. Cohen’s D values were calculated as effect estimates for unpaired *t* tests. Pearson’s correlation test or multiple regression was used to assess the relationships between the number of BCAA-increasing alleles and the CSA of fast-twitch and slow-twitch muscle fibers (adjusted for age, training frequency, and meat and dairy intake). All data are presented as mean (SD). *p* values < 0.05 were considered statistically significant.

## 3. Results

### 3.1. Hardy–Weinberg Equilibrium (HWE) and Genotype Distribution

Genotype distributions across both groups for each SNP are described in Table 3. All were in HWE (χ^2^ ≤ 1.661, *p* ≥ 0.197). There were no differences in genotype frequency between groups (*p* ≥ 0.256). There were no differences in the sum of the BCAA-increasing alleles between groups (WPS: 7.1 (1.2) alleles; Non-WPS: 7.3 (1.3) alleles; *p* = 0.468).

### 3.2. Anthropometry

Body mass (*p* = 0.009; Cohen’s D =0.710) and BMI (*p* = 0.01; Cohen’s D =0.712) were significantly lower in WPS than Non-WPS (Table 2). In participants with body composition data (*n* = 39), fat mass (*p* = 0.005; Cohen’s D = 1.09) and body fat percentage (*p* = 0.006; Cohen’s D =1.05) were significantly lower, and muscle mass significantly higher (*p* = 0.01; Cohen’s D =1.00), in WPS than Non-WPS (Table 2).

### 3.3. Muscle Fiber Distribution and Cross-Sectional Area (CSA)

Fiber type distribution did not differ between WPS and Non-WPS (STMF, *p* = 0.138; FTMF, *p* = 0.066; Table 2). The CSA of all fibers did not differ between WPS and Non-WPS (*p* = 0.755). Similarly, there were no differences between groups for the CSA of STMF (*p* = 0.801) or FTMF (*p* = 0.467) (Table 2). 

### 3.4. Polygenic Association with Muscle Fiber CSA

**Figure 1:**
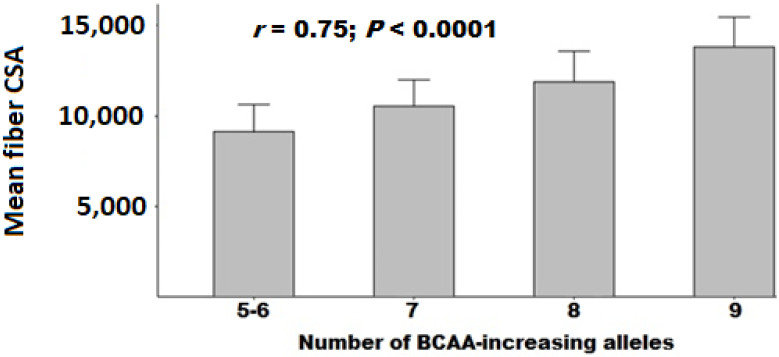
Mean all (slow-twitch + fast-twitch) muscle fiber CSA (μm^2^) in participants consuming WPS according to number of BCAA-increasing alleles.

The number of BCAA-increasing alleles ranged from 5 to 10 alleles in the Non-WPS group and from 5 to 9 alleles in the WPS group. No correlation was found between the number of BCAA-increasing alleles and the CSA of all fibers in the Non-WPS group (*r* = 0.01, *p* = 0.943). There remained no correlation after correction for covariates (age, physical activity, and meat and dairy intake; *p* = 0.961). However, the number of BCAA-increasing alleles was strongly correlated with the CSA of all muscle fibers in the WPS group (*r* = 0.75, *p* < 0.0001, Figure 1). 

This correlation remained statistically significant after correction for covariates (age, physical activity, and meat and dairy intake; *p* = 0.0008), and the relationship was stronger for FTMF (CSA in carriers of 5–6 alleles: 4512 (1145) μm^2^; 7 alleles: 5436 (474) μm^2^; 8 alleles: 6405 (1363) μm^2^; and 9 alleles: 7634 (2099) μm^2^; *p* = 0.001) than STMF (CSA in carriers of 5–6 alleles: 4612 (959) μm^2^; 7 alleles: 5094 (1073) μm^2^; 8 alleles: 5462 (490) μm^2^; and 9 alleles: 6159 (1367) μm^2^; *p* = 0.048). Correlations for all muscle fibers and FTMF in the WPS group remained significant after correction for multiple testing (i.e., *p* = 0.05/6 tests = 0.0083).

## 4. Discussion

This study investigated the polygenic contribution of SNPs previously associated with BCAA metabolite levels on muscle fiber CSA in participants with and without WPS. The main finding was the positive correlation of BCAA-increasing alleles with muscle fiber CSA in endurance-trained participants consuming WPS, particularly for fast-twitch fibers. However, the number of BCAA-increasing alleles was unrelated to fiber CSA in Non-WPS participants, suggesting that the polygenic relationship of these SNPs with fiber CSA depends on exogenous WPS as a source of BCAAs. We also observed that body mass, BMI, and fat percentage were lower, and muscle mass higher, in participants consuming WPS, supporting previous associations of BCAAs with fat-free mass. Our findings indicate that carrying a higher number of BCAA-increasing alleles may enhance the capacity to metabolise BCAAs from WPS, augmenting MPS and contributing to greater fiber CSA.

In participants consuming WPS, the collective number of alleles previously associated with increased BCAA levels was positively correlated with fiber CSA (*PPM1K* rs1440580 A, *APOA5* rs2072560 C, *CBLN1* rs1420601 C, *DDX19B* rs12325419 G, and *TRMT61A* rs58101275 G alleles). Previously, BCAA-increasing alleles were associated with T2D risk phenotypes in sedentary populations [18]. To our knowledge, this study describes the first polygenic association of BCAA-increasing alleles with fiber CSA when combined with WPS, suggesting these variants influence WPS efficiency. Metabolomic data link BCAA-increasing alleles with impaired BCAA catabolism, potentially explaining their association with T2D risk [18]. However, we observed greater fiber CSA in WPS participants with more BCAA-increasing alleles. This suggests that, for active individuals, catabolism and utilisation of BCAAs from WPS are in fact enhanced by BCAA-increasing alleles. Mechanistically, it is possible that higher BCAA catabolism pathway mRNA expression in active individuals [14] is complemented by BCAA-increasing alleles. Consequently, carriers of multiple alleles may have better capacity to metabolise the circulating BCAAs ingested through WPS [10], increasing MPS [3] and contributing to greater fiber CSA. Further study of the genomic and metabolomic relationship between these SNPs, BCAA catabolism, and MPS is required to support this novel hypothesis.

The contrast between our proposed mechanistic link of BCAA-increasing alleles with improved BCAA catabolism and previous associations of BCAA-increasing alleles with impaired BCAA catabolism [18] may be explained by different study populations. Previous investigations involved sedentary and/or T2D populations [18], and ours physically active participants. Recent physical activity acutely reduces BCAA levels [15], and twin studies report lower BCAA levels in chronically active versus non-active twins [13]. Consequently, acute and chronic reductions in BCAA levels appear to be mediated by exercise, supported by higher mRNA expression of BCAA catabolic pathways in active individuals [14]. Together, these studies indicate that exercise is an important regulator of BCAA catabolism. In conjunction with exogenous WPS, we suggest that exercise stimuli combined with a heritable predisposition for increased BCAA metabolism leads to favourable physiological outcomes, such as an enhanced capacity to maintain or increase muscle mass. Indeed, animal models demonstrate that both leucine supplementation and moderate aerobic exercise inhibit ubiquitin proteosome activity and increase the activity of mTOR and its downstream targets ribosomal protein S6 kinase (p70S6K) and eukaryotic translation initiation factor 4E-binding protein 1 (4E-BP1) [22]. The effect of these signals on phenotypes including fiber CSA was more pronounced when aerobic exercise and leucine were combined [22]. Accordingly, we suggest that participants in the present study with a higher number of BCAA-increasing alleles have better capacity to catabolise BCAAs and stimulate MPS, and that the lack of relationship between these alleles and fiber CSA in Non-WPS participants is due to the absence of WPS-derived BCAAs. The positive correlation between BCAA-increasing alleles and fiber CSA suggests a dose–response relationship for the efficiency to catabolise and utilise BCAAs from WPS for MPS, particularly in FTMF. Previously, Peroxisome Proliferator Activated Receptor γ (*PPARG*) [23] and Ubiquitin Protein Ligase E3 Component *N*-Recognin 5 (*UBR5*) [24] gene SNPs were associated with FTMF CSA, with the number of testosterone or strength-associated alleles also correlating positively with this phenotype [25,26]. Consequently, it appears that FTMF CSA is in part genetically influenced, with the present study suggesting this polygenic trait might involve variants affecting BCAA metabolism.

Under restricted energy, FTMF are more susceptible to protein degradation than STMF [27], suggesting the effectiveness of BCAAs from WPS may be pronounced in FTMF. In endurance athletes, circulatory BCAA levels decrease during competition [28] contributing to muscle damage and proteolysis, which impede performance and recovery [29]. Consequently, competitors commonly ingest WPS to enhance recovery and maintain muscle mass [30]. In this study, the correlation between total BCAA-increasing alleles and FTMF CSA was stronger than for STMF. Our findings are in line with the previous study showing that even low-intensity training induces more hypertrophic changes in fast-twitch than in slow-twitch muscle fibers [31]. These differences may be due to the different levels of sensitivity of fast and slow muscle fibers to anabolic stimuli. We propose that carrying more BCAA-increasing alleles improves the efficiency of BCAAs ingested via WPS by inhibiting ubiquitin proteosome activity, increasing the activity of mTOR and its downstream targets p70S6K and 4E-BP1, and offsetting protein degradation, particularly in FTMF. Whilst potentially explaining the positive correlation between BCAA-increasing alleles and FTMF CSA in WPS participants, mechanistic studies should determine how the SNPs investigated in this study interact, and their consequent effects on BCAA catabolism, MPS, and fiber phenotypes.

The individual role of each investigated SNP within skeletal muscle is poorly understood. The Protein Phosphatase Mg2+/Mn2+ Dependent 1K (*PPM1K*) rs1440580 SNP has been associated with whole-body fat-free mass (UK Biobank), with the A allele associated with increased metabolites of all three BCAAs. Other *PPM1K* SNPs have been associated with maple syrup urine disease, characterised by the inability to metabolise BCAAs due to branched-chain ketoacid dehydrogenase deficiency [32]. *PPM1K* SNPs are also associated with circulating BCAA levels in relation to Alzheimer’s [33] and T2D [18]. The Apolipoprotein A5 (*APOA5*) rs2072560 SNP has been associated with IGF1 levels (UK Biobank) and the C allele with increased valine metabolites. This SNP was also associated with dyslipidemia [34] and circulating triglycerides [35]. Additional *APOA5* SNPs are associated with lipid levels [35], metabolic syndrome [36] and statin treatment efficacy [37]. The Cerebellin 1 Precursor (*CBLN1*) rs1420601 SNP is associated with whole-body fat-free mass and isoleucine levels (UK Biobank), as well as with impaired BCAA metabolism and T2D risk [18]. The influence of this variant on isoleucine levels was also linked to Alzheimer’s risk [33]. Literature concerning the DEAD-Box Helicase 19B (*DDX19B*) rs12325419 SNP is limited, though there are links with BMI and leucine metabolism [19]. Another SNP near to *DDX19B* was associated with leucine levels, but was not among a collection of variants related to Alzheimer’s disease [33]. Like *CBLN1* rs1420601, the TRNA Methyltransferase 61A (*TRMT61A*) rs5810275 SNP is associated with isoleucine levels [18] and whole-body fat-free mass [19], with increased isoleucine levels linked to this SNP associated with Alzheimer’s risk [33]. Currently, evidence linking these SNPs to muscle phenotypes is limited. Nevertheless, all appear to influence the levels of one or more BCAA metabolites, suggesting each might influence BCAA metabolism, which is critical for MPS [1]. We suggest that a high number of BCAA-increasing alleles improves the efficiency of WPS by enhancing BCAA metabolism, MPS stimulation, and contributing to greater fiber CSA.

Participants habitually consuming WPS had lower body mass and BMI than their Non-WPS counterparts and in a sub-sample of participants (*n* = 39), WPS had relatively greater muscle mass than Non-WPS, the latter with higher absolute and relative quantities of fat mass. Supplementation with BCAAs is shown to induce significant, preferential reductions in visceral adipose tissue [38], which might explain a lower relative fat mass in WPS participants despite similar training frequency between groups. Height did not differ between groups, meaning that the higher BMI of Non-WPS participants is explained by differences in body mass. These observations, regardless of genetic variability, support previous literature [9] and suggest WPS consumption in endurance-trained individuals promotes the maintenance of skeletal muscle. 

This study presents novel data of a positive polygenic relationship between BCAA-increasing alleles and muscle fiber CSA. However, there are also limitations. Firstly, data regarding protein intake were retrospective, meaning we cannot guarantee the exact protein intake of WPS participants. Second, this study did not measure BCAA metabolite levels and other laboratory data, meaning we cannot yet prove whether these alleles do increase BCAA levels in this population, and whether this may relate to fiber CSA. We also recognise the modest samples size in the present study, and encourage independent replication in larger cohorts with a prospective study design (for example a randomized, double-blind, and longitudinal design in response to resistance/endurance training) to tightly control dietary protein intake. 

## 5. Conclusions

In conclusion, we have identified a positive correlation of BCAA-increasing alleles with muscle fiber CSA in endurance-trained participants consuming WPS. These novel findings add to the existing literature from clinical populations by providing new evidence to suggest the capacity for BCAA catabolism in physically active participants is genetically influenced. 

## Figures and Tables

**Table 1:** SNPs associated with plasma BCAA levels (literature data).

SNP	Gene	Metabolite	Increasing Allele	*p* Value	Reference	Other Traits (UK Biobank)
rs1440580	*PPM1K*	Valine	A	1.3 × 10^−^^31^	[19]	Whole body fat-free mass (*p* = 0.011)
		Leucine		1.9 × 10^−^^24^		
		Isoleucine		1.7 × 10^−^^14^		
rs12325419	*DDX19B*	Leucine	G	4.5 × 10^−^^8^	[19]	BMI (*p* = 2.1 × 10^−^^7^)
rs2072560	*APOA5*	Valine	C	3.3 × 10^−^^9^	[19]	IGF1 levels (*p* = 0.025)
rs58101275	*TRMT61A*	Isoleucine	G	2.78 × 10^−^^8^	[18]	Whole body fat-free mass (*p* = 3.7 × 10^−^^7^)
rs1420601	*CBLN1*	Isoleucine	C	3.71 × 10^−^^8^	[18]	Whole body fat-free mass (*p* = 0.0033)

**Table 2:** Participant characteristics according to whey protein supplementation (WPS) group.

Characteristics	Non-WPS	WPS	*p* Value
	*n* = 53	*n* = 22	
Height (m)	1.81 (6.24)	1.79 (5.72)	0.370
Weight (kg)	79.5 (8.7)	73.8 (7.3)	0.009 *
BMI (kg/m^2^)	24.4 (2.2)	23.0 (1.7)	0.01 *
Age (y)	32.1 (8.7)	31.5 (8.3)	0.779
FTMF CSA (μm^2^)	5367 (1321)	5634.10 (1691)	0.467
STMF CSA (μm^2^)	5237 (1302)	5157.35 (1099)	0.801
All Fiber CSA (μm^2^)	10,604 (2377)	10,791.41 (2277)	0.755
FTMF (%)	53.1 (17.0)	45.5 (13.8)	0.066
STMF (%)	50.6 (17.7)	56.9 (13.9)	0.138
Physical activity (sessions)	6.0 (4.5)	7.7 (3.8)	0.078
Frequency of meat intake	5.0 (1.9)	5.8 (1.5)	0.121
Frequency of dairy intake	4.2 (2.2)	4.1 (2.3)	0.879
	(*n* = 25)	(*n* = 14)	
Fat mass (kg)	16.0 (5.3)	11.4 (2.8)	0.005 *
Fat percentage (%)	20.2 (5.4)	15.6 (3.0)	0.006 *
Muscle mass (kg)	42.9 (3.9)	46.0 (2.0)	0.01 *

* *p* < 0.05, statistically significant differences between two groups. Data presented as mean (standard deviation). FTMF, fast-twitch muscle fibers. STMF, slow-twitch-muscle fibers. Body composition analysis was performed in a sub-sample of participants (*n* = 39).

**Table 3:** Genotype distribution for all SNPs.

SNP	Genotype	Non-WPS	WPS
*PPM1K*	TT	14 (26.4)	5 (22.7)
rs1440580	TA	28 (52.8)	10 (45.5)
	AA	11 (20.8)	7 (31.8)
	MAF	0.472	0.545
*APOA5*	CC	47 (88.7)	20 (90.9)
rs2072560	CT	6 (11.3)	2 (9.1)
	TT	0 (0.0)	0 (0.0)
	MAF	0.057	0.045
*CBLN1*	TT	13 (24.5)	7 (31.8)
rs1420601	TC	28 (52.8)	12 (54.5)
	CC	12 (22.6)	3 (13.6)
	MAF	0.491	0.409
*DDX19B*	GG	39 (73.6)	16 (72.7)
rs12325419	GA	13 (24.5)	6 (27.3)
	AA	1 (1.9)	0 (0.0)
	MAF	0.142	0.136
*TRMT61A*	GG	41 (77.4)	13 (59.1)
rs58101275	GA	10 (18.9)	8 (36.4)
	AA	2 (3.8)	1 (4.5)
	MAF	0.132	0.227

Data are number of participants (% of group). MAF, minor allele frequency.

## Data Availability

The data presented in this study are available on request from the corresponding author.
